# Is intraoperative frozen section analysis of the proximal bile ducts in hilar cholangiocarcinoma of limited value?

**DOI:** 10.1002/cam4.920

**Published:** 2016-09-29

**Authors:** Wen‐Jie Ma, Anuj Shrestha, Fu‐Yu Li

**Affiliations:** ^1^ Department of Hepatobiliary SurgeryWest China Hospital of Sichuan UniversityChengduChina; ^2^ Department of General SurgeryGandaki Medical CollegePokharaNepal

**Keywords:** hilar cholangiocarcinoma, frozen section

## Abstract

Mantel et al. showed that the use of intraoperative frozen section analysis of the proximal bile ducts has a limited contribution in obtaining secondary R0 resections and final resection status had no impact on recurrence rate in hilar cholangiocarcinoma. However, the accuracy, sensitivity, and specificity of intraoperative frozen section analysis were determined by the specific pathologic features of the tumor and the different experienced pathologists in different pathology laboratories. It has been demonstrated that tumor‐free resection margin (R0) is the most prognostic factor for survival, as well as the only factor that can be modified by the surgeons. Ribero et al. reported an improvement in prognosis was found in the secondary R0 group. As the conclusion given by Mantel et al. and Shingu et al., which is contrary to Ribero et al. Before the real role of intraoperative frozen section in the analysis of the margin of proximal bile ducts in treating hilar cholangiocarcinoma is concluded, further studies are still needed.

In a retrospective clinical study investigating the accuracy and consequences of intraoperative frozen section (FS) of the proximal bile duct margins in patients with hilar cholangiocarcinoma (HCCA), Mantel et al. 1 showed that the use of intraoperative frozen section analysis of the proximal bile ducts has a limited contribution in obtaining secondary R0 resections and final resection status had no impact on recurrence rate. Many aspects of this study were well done. The authors chose the sensitivity, false‐negative rate, and recurrence rate to explain their argument. Other than a number of study subjects and a consistent intraoperative frozen section analysis, the factors affecting long‐term survival were also demonstrated by univariable analysis, such as lymph node metastases and final resection status.

In this study, in almost one‐third of HCCA cases, FS did not detect tumor cells at the resection margin and the false‐negative rate of 16% which leads to an additional resection was erroneously withheld in eight patients. Even though the intraoperative assessment of bile duct margin clearance is a useless prognostic marker for patients undergoing resection for HCCA in few reports, frozen section analysis of bile duct margins is the most used method to guide the extent of surgical resection for HCCA. 2 The accuracy, sensitivity, and specificity of 89, 68, and 97%, respectively, of intraoperative FS in this study are higher than those of 56.5, 75.0, and 46.7% reported by Okazaki et al. 3 except sensitivity. It suggests us that these are unlikely to be totally objective, besides the specific pathologic features of the tumor, these are determined by different experienced pathologists in different pathology laboratories. Furthermore, as technology advances, the accuracy, sensitivity, and specificity of FS would become more accuracy.

We noted that FS contributed to secondary R0 resections was low in this study (only three patients), and the low rates of secondary obtained R0 resections (4–9%) reported by Lee et al., and Endo et al. Further resection of the bile duct at the proximal side is technically difficult due to encroachment onto vital structures and adjacent liver parenchyma. Shingu et al. 6 reported that further resection does not contribute to improvement in survival. The opposite of this is an improvement in prognosis was found in the secondary R0 group reported by Ribero et al.^7^ However, it has been demonstrated that tumor‐free resection margin (R0) is the most prognostic factor for survival, as well as the only factor that can be modified by the surgeons 2. For patient who can be further resected, 54 to 83% can achieve R0 resection 2. The reason of additional resection does not improve the survival probably lies in the limited additional resection of a margin‐positive proximal bile duct <5 mm 6. In their article, Mantel et al. reported that the length of the additional resections in the three remaining patients was >5 mm. Unfortunately, their numbers were not large enough to allow a survival analysis comparing patients with a secondary obtained R0 resection to those with an R1 or primary R0 resection. As the conclusion given by Mantel et al. and Shingu et al., which is contrary to Ribero et al. We argue that before the real role of intraoperative frozen section in the analysis of the margin of proximal bile ducts in treating hilar cholangiocarcinoma is concluded, further studies are still needed.

## Conflict of Interest

No benefits in any form have been received or will be received from a commercial party related directly or indirectly to the subject of this article.
